# *Helicobacter pylori*-induced gastric cancer is orchestrated by MRCKβ-mediated Siah2 phosphorylation

**DOI:** 10.1186/s12929-021-00710-0

**Published:** 2021-02-03

**Authors:** Pragyesh Dixit, Shrikant B. Kokate, Indrajit Poirah, Debashish Chakraborty, Duane T. Smoot, Hassan Ashktorab, Niranjan Rout, Shivaram P. Singh, Asima Bhattacharyya

**Affiliations:** 1 School of Biological Sciences, National Institute of Science Education and Research (NISER) Bhubaneswar, HBNI, P.O. Bhimpur-Padanpur, Via Jatni, Khurda, 752050 Odisha India; 2 Department of Medicine, Meharry Medical Center, Nashville, TN 37208 USA; 3 Department of Medicine, Howard University, Washington, DC 20060 USA; 4 Department of Pathology, Acharya Harihar Post Graduate Institute of Cancer, Cuttack, 753007 Odisha India; 5 Department of Gastroenterology, SCB Medical College, Cuttack, 753007 Odisha India; 6 Present Address: Institute of Biotechnology, University of Helsinki, P.O. Box 56, 0014 Helsinki, Finland

**Keywords:** Cdc42BPB, E3 ubiquitin ligase, Gastric cancer, *Helicobacter pylori*, Host–pathogen interaction, Proteasome

## Abstract

**Background:**

*Helicobacter pylori*-mediated gastric carcinogenesis is initiated by a plethora of signaling events in the infected gastric epithelial cells (GECs). The E3 ubiquitin ligase seven in absentia homolog 2 (Siah2) is induced in GECs in response to *H. pylori* infection. Posttranslational modifications of Siah2 orchestrate its function as well as stability. The aim of this study was to evaluate Siah2 phosphorylation status under the influence of *H. pylori* infection and its impact in gastric cancer progression.

**Methods:**

*H. pylori*-infected various GECs, gastric tissues from *H. pylori*-infected GC patients and *H. felis*-infected C57BL/6 mice were evaluated for Siah2 phosphorylation by western blotting or immunofluorescence microscopy. Coimmunoprecipitation assay followed by mass spectrometry were performed to identify the kinases interacting with Siah2. Phosphorylation sites of Siah2 were identified by using various plasmid constructs generated by site-directed mutagenesis. Proteasome inhibitor MG132 was used to investigate proteasome degradation events. The importance of Siah2 phosphorylation on tumorigenicity of infected cells were detected by using phosphorylation-null mutant and wild type Siah2 stably-transfected cells followed by clonogenicity assay, cell proliferation assay, anchorage-independent growth and transwell invasion assay.

**Results:**

Siah2 was phosphorylated in *H. pylori*-infected GECs as well as in metastatic GC tissues at residues serine^6^ (Ser^6^) and threonine^279^ (Thr^279^). Phosphorylation of Siah2 was mediated by MRCKβ, a Ser/Thr protein kinase. MRCKβ was consistently expressed in uninfected GECs and noncancer gastric tissues but its level decreased in infected GECs as well as in metastatic tissues which had enhanced Siah2 expression. Infected murine gastric tissues showed similar results. MRCKβ could phosphorylate Siah2 but itself got ubiquitinated from this interaction leading to the proteasomal degradation of MRCKβ and use of proteasomal inhibitor MG132 could rescue MRCKβ from Siah2-mediated degradation. Ser^6^ and Thr^279^ phosphorylated-Siah2 was more stable and tumorigenic than its non-phosphorylated counterpart as revealed by the proliferation, invasion, migration abilities and anchorage-independent growth of stable-transfected cells.

**Conclusions:**

Increased level of Ser^6^ and Thr^279^-phosphorylated-Siah2 and downregulated MRCKβ were prominent histological characteristics of *Helicobacter*-infected gastric epithelium and metastatic human GC. MRCKβ-dependent Siah2 phosphorylation stabilized Siah2 which promoted anchorage-independent survival and proliferative potential of GECs. Phospho-null mutants of Siah2 (S6A and T279A) showed abated tumorigenicity.

## Background

GC progresses through multiple stages and involves various factors. *H. pylori* (a class I carcinogen) is the prime factor for increased risk of GC. This pathogen infects human stomach and acquires a number of adaptations to survive in its harsh ecological niche, the pylorus part of the human stomach. Infection of *H. pylori* initiates various pathophysiological events in the gastric epithelium which promote GC [1–4].

Siah proteins belonging to the E3 ubiquitin ligase family are associated with several cancers including GC [5–7]. *H. pylori* infection upregulates Twist-related protein 1 and E26 transformation-specific sequence 2 which transcriptionally increase Siah2 level. Increase in Siah2 aids in *H. pylori*-mediated GC progression [8]. Siah2 enhances invasiveness and progression of GC through the regulation of degradation of its various binding partners [9]. Siah2 function and subcellular localization are modulated by post-translational modifications. Acetylation of Siah2 promotes Siah2 stabilization in GECs infected with *H. pylori* and induces Siah2-mediated prolyl hydroxylase 3 degradation, thus contributing to infection-induced hypoxia [7]. Siah2 phosphorylation by p38 mitogen-activated protein kinase and dual-specificity tyrosine phosphorylation-regulated 2 kinases play critical roles in hypoxic stress response by regulating prolyl hydroxylase 3 [10, 11]. Phosphorylation of Siah2 by Src kinase transforms breast cancer by degrading CCAAT/enhancer-binding protein delta, a transcription factor known for its tumor-suppressive functions [12]. p53 kinase homeodomain-interacting protein kinase 2 is also known to phosphorylate Siah2, thus rendering it more active and resulting in its ubiquitination followed by degradation. This further leads to the downregulation of p53 [13]. Serine-threonine checkpoint kinase 2-mediated Siah2 phosphorylation leads to checkpoint kinase 2 downregulation having consequences in DNA damage response and cell cycle control [14]. However, the status and mechanism of Siah2 phosphorylation and its importance in *H. pylori*-mediated GC remain elusive.

Keeping in purview the crucial role of Siah2 proteins in GC progression, we studied Siah2 phosphorylation status in the *H. pylori*-infected gastric epithelium and investigated the effect of phospho-Siah2 in GC progression. Here we report that *H. pylori* infection enhances Siah2 phosphorylation through myotonic dystrophy kinase-related Cdc42-binding kinase β (MRCKβ), a Ser/Thr kinase. MRCKβ remains anchored by a tight junction protein zonula occludens-1 at the leading-edge of the invasive cell and controls migration [15]. Squamous cancer cell migration [16] and ErbB2-driven breast cancer invasion [17] are MRCKβ-mediated. MRCKβ has also been implicated to play a role in skin cancer progression [18] and HeLa cell elongation [19]. This study identifies that MRCKβ-mediated Siah2 phosphorylation at Ser^6^ and Thr^279^ increases Siah2 stability and positively contributes in the GC progression. The presence of phosphorylated Siah2 in the human GC biopsy tissues also reveal its potential diagnostic and therapeutic importance.

## Methods

### Cells, *Helicobacter* strains and reagents

MKN45 and AGS cells (human gastric epithelial cell lines), *cag* PAI positive *H. pylori strain* 26695, *cag* PAI negative *H. pylori* strain 8–1 and *H. felis* strain 49179 were purchased from the American Type Culture Collection (Manassas, VA). Immortalized non-neoplastic HFE145 GECs, other mentioned cell lines and *H. pylori* were maintained as per established protocols [7, 20]. Cells were infected with *H. pylori* using various multiplicity of infection (MOI) for the indicated durations. Proteasomal inhibitor MG132 or Z-Leu-Leu-Leu-al (Millipore-Sigma, St. Louis, MO) at 50 µM concentration was used in assays aiming to identify involvement of proteasomal degradation. Gentamicin (Himedia, Nashik, India) was used in clonogenicity assay to kill extracellular *H. pylori* at 150 μg/ml concentration for 3 h.

### Expression plasmids and site-directed mutagenesis

Full-length human *siah2* (Origene Technologies, MD; hereafter referred to as *siah2* WT) and eukaryotic expression vector pcDNA3.1+ (Thermo Fisher Scientific, MA) were purchased. Various *siah2* mutants were generated from the *siah2* WT constructs by site-directed mutagenesis (QuikChange site-directed mutagenesis kit, Agilent Technologies, CA) using suggested procedure. Primers used were as follows:

For *siah2* S6A: 5′ CATGAGCCGCCCGTCCGCCACCGGCCCCAGCGC 3’.

For *siah2* T275A: 5′ GGGAACCGGCGGAGATTGGCCTGGGAGGCCACGCCCC 3’.

For *siah2* T279A: 5′ AGATTGACCTGGGAGGCCGCGCCCCGTTCGATTCATG 3’.

For *siah2* S282A: 5′ TGGGAGGCCACGCCCCGTGCGATTCATGACGGTGTGG 3’.

pEGFP-N1-Cdc42BPB (*mrckβ*) was gifted by Prof. Naoki Mochizuki, National Cerebral and Cardiovascular Center, Osaka, Japan.

### Transient transfection and generation of stable cell lines

24 h post-plating and 1 h prior to transfection, spent media was discarded and replenished by 10% FBS (HiMedia) containing RPMI1640 media (HiMedia). Cells were transfected with DNA, P3000 reagent and Lipofectamine 3000 (Invitrogen, CA) as per the company’s instructions. When required, cells were transfected with control siRNA (Santa Cruz Biotechnology, TX) and *mrckβ* siRNA (Santa Cruz Biotechnology) using Lipofectamine 3000. Similar protocol was used for transfection of control siRNA (Origene) and siRNA of *siah2* (Origene). G418 solution (Millipore-Sigma) was used to select stably-transfected clones following standard protocol.

### Western blotting and coimmunoprecipitation assays

Western blotting was performed following standard protocols [7, 9, 20]. Briefly, total cell lysates were prepared from GECs (with or without *H. pylori* infection), run on SDS-PAGE and electro-transferred onto PVDF membrane. MRCKβ and Siah2 (Santa Cruz Biotechnology) Phospho-Serine (Millipore-Sigma), Phospho-Threonine (Cell Signaling Technology, MA), Ubiquitin (Cell Signaling Technology) and GAPDH (Abgenex, Bhubaneswar, India) primary antibodies were used to probe electro-blotted membranes. Customized human P-Ser^6^-Siah2 and P-Thr^279^-Siah2 antibodies were generated (Bioklone Biotech Pvt. Ltd., Chennai, India). Immunoblots were detected by Thermo Scientific SuperSignal West Femto Chemiluminescent Substrate kit (Thermo Fisher Scientific, IL) and images were captured using BioRad Chemidoc XRS+. Densitometric analysis was done using ImageLab software (Bio-Rad, CA). Whole-cell lysates were immunoprecipitated using Siah2 antibody and immunoprecipitates were detected by western blotting.

### Immunofluorescence microscopy and image analysis

GECs were cultured on coverslips, infected with *H. pylori* (MOI 200, 12 h) and processed for immunofluorescence microscopy. Cells were incubated with desired specific antibodies at 4 °C for overnight using manufacturer’s recommended dilution. Fluorophore Alexa Fluor-488 and 594-tagged secondary antibodies (Invitrogen, OR) were used. 4′, 6-Diamidino-2-phenylindoledihydrochloride (DAPI; Invitrogen) was used for 20 min to stain nuclei. Digital images were taken by Nikon microscope (Eclipse Ti-U, Nikon, Tokyo, Japan) fitted with the digital monochrome camera DS Qi2 (Nikon).

Confocal images were captured on Leica DMi8 confocal microscope (Leica, Germany) using Leica Application Suite X software. Captured images were processed and analysed using NIS Advanced Research software (Nikon).

### Human gastric biopsy and mouse gastric tissue collection

Metastatic stage IV antral gastric adenocarcinoma biopsy samples were obtained from the gastric antrum of urease-test positive GC patients (n = 9). A noncancerous biopsy sample from the adjacent area was also obtained from each patient which was used as a paired-control. A prior-informed signed consent was obtained for all patients and patient privacy was preserved. Patients who were treated for *H. pylori* eradication were not included in the study. The protocol used for biopsy collection was approved by the Institutional Ethics Committee for Human Research, National Institute of Science Education and Research and was in compliance with the Helsinki Declaration (2013), World Medical Association.

7–8 weeks old C57BL/6 mice (procured from National Centre for Laboratory Animal Sciences of the National Institute of Nutrition, Hyderabad, India) were blindly divided into uninfected (n = 16) and infected (n = 16) irrespective of their sex and were infected with *H. felis* or 1X PBS following a standard protocol [21]. After 18 months of infection, mice were euthanized and their stomachs were collected. This study was approved by the Institutional Animal Ethics Committee of National Institute of Science Education and Research. Samples were fixed using 4% paraformaldehyde and sectioned at 5 μm thickness using a cryomicrotome.

### MTT assay

Proliferation of pcDNA3.1+, *siah2* WT or *siah2* phospho-null mutant (*siah2* S6A and *siah2* T279A) stable-transfected MKN45 cells infected with 200 MOI *H. pylori* for 12 h was assessed using a MTT assay kit (EZcount MTT cell assay kit, Himedia) following the company’s recommended protocol.

### Soft agar assay

Stably-transfected MKN45 cells were used to assess anchorage-independent growth following our previously-established protocols [8, 9].

### Clonogenic survival assay

pcDNA3.1+, WT, S6A and T279A *siah2*-expressing MKN45 stable cells were seeded in 24-well cell culture dishes with a density of 16.6 × 10^4^ followed by infection for 12 h at 200 MOI. Next, extracellular pathogens were killed by using 150 μg/ml gentamicin for 3 h. Cells were trypsinized followed by seeding in 60 mm^2^ dishes with 1000 cells/dish. After 3 weeks of incubation, cells were fixed with 4% paraformaldehyde. After fixing and staining with crystal violet (0.5%), the number of colonies formed were counted.

### In vitro invasion assay

pcDNA3.1+, WT, S6A and T279A *siah2*-expressing AGS stable cells were seeded on cell culture inserts with 0.3 cm^2^ polyethylene terephthalate membrane (8-μm pore size and 6.4-mm diameter; Falcon Corning, MA) coated with growth factor–reduced standard Matrigel matrix (Becton Dickinson, NJ) and further processed according to our previously reported protocol [9, 22]. Images were captured from at least three different fields using an inverted microscope as already mentioned. Invaded cells were counted.

### Cell population doubling assay

pcDNA3.1+, WT, S6A and T279A *siah2*-expressing MKN45 stable cells were seeded in 24 well plate at a density of 16.6 × 10^4^ cells/well. After 24 h of incubation, cells were infected with 200 MOI of *H. pylori* for 12 h. 150 μg/ml gentamicin was used for 3 h to kill extracellular bacteria. Next, cells were trypsinized and 10,000 cells were seeded in 24 well plate. After seeding, the number of cells were counted on day 2, 3 and 4. The population doubling (PD) was calculated using the formula below [23, 24] where Ns and Nh are the number of seeded and harvested cells, respectively:

$${\text{PD}} = \left[ {{\text{log}}\left( {{\text{Nh}}} \right) - {\text{log}}\left( {{\text{Ns}}} \right)} \right]/{\text{log 2}}$$

The cumulative population doubling (cPD) level was calculated by summation of the PD values obtained for each day and represented as graphs with cPD at y-axis [25].

### Statistical analysis

GraphPad Prism 7.00 software (GraphPad, CA) was used for statistical analyses. Values represent mean ± sem. *P* < 0.05 determined statistical significance using either one-way or two-way ANOVA followed by Tukey’s post hoc test. All results represent at least three independent experimental repeats.

## Results

### *H. pylori* infection enhances Siah2 phosphorylation

**Fig. 1:**
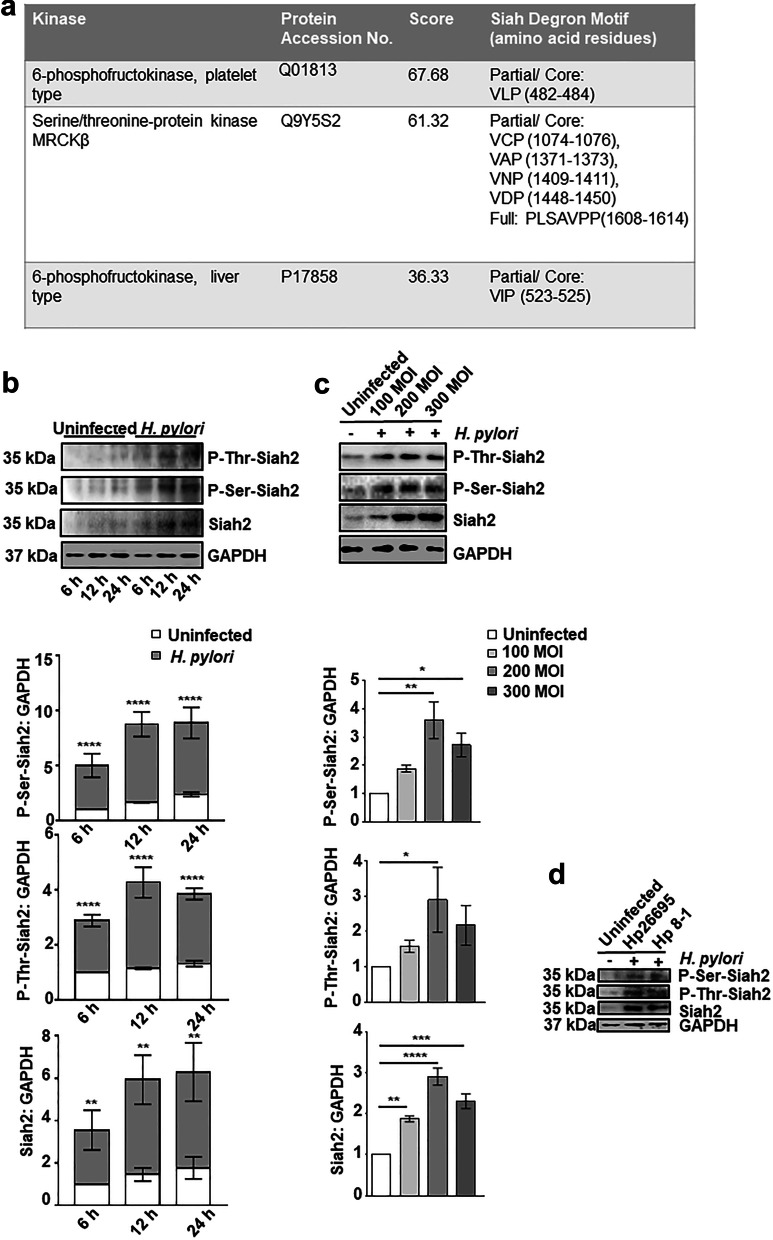
Siah2 phosphorylation is increased in *H. pylori*-infected GEC. **a** Table showing partial or full Siah degron motifs in the protein sequence of kinases identified by mass spectrometry. **b** Western blotting of total cell extracts prepared from the uninfected and *H. pylori*-infected (200 MOI) MKN45 cells at 6, 12, and 24 h post-infection show increased P-Ser-Siah2, P-Thr-Siah2 and Siah2 levels. Histograms clearly represent a significant increment of P-Ser-Siah2, P-Thr-Siah2 and Siah2 at all time points. Statistical significance is determined by two-way ANOVA followed by Tukey’s post hoc analysis. **c** Western blot of whole cell lysate from MKN45 cells uninfected and infected with *H. pylori* (MOI 100, 200 and 300 MOI for 12 h). Graphical representation reveals a significant increase of P-Ser-Siah2, P-Thr-Siah2 and Siah2 with 200 MOI of infection. One-way ANOVA is used to determine statistical significance. **d** A representative western blot from uninfected and infected (200 MOI for 12 h of *H. pylori* 26695 strain [*cag* PAI +] or 8–1 strain [*cag* PAI −]) MKN45 cells representing P-Ser-Siah2, P-Thr-Siah2 and Siah2. GAPDH is used as a loading control. All graphical data are mean ± sem (n = 3). *P < 0.05, **P < 0.01, ***P < 0.001, ****P < 0.0001

Our group identified that Siah2 was optimally induced by *H. pylori* 12 h after infection and at 200 MOI [7, 9]. In order to identify kinases that interact with Siah2 and to enrich interaction signals, overexpression-immunoprecipitation assay was performed. MKN45 cells were transfected with either *siah2* overexpression plasmid or the vector control followed by *H. pylori* 26695 (*cag* PAI + strain) infection for 12 h at 200 MOI. Whole cell lysates were subjected to immunoprecipitation with Siah2 antibody and immunocomplexes were separated by SDS-PAGE followed by staining of the gel with Coomassie brilliant blue (R-250). Bands were cut and mass spectrometry was done. Among the identified kinases from the Siah2-immunocomplex was MRCKβ, a Ser/Thr kinase and two isoforms of 6-phosphofructokinase (6-PFK). Our analysis further revealed that MRCKβ has four partial and one full Siah-degron motifs but 6-PFK isoforms have only one partial degron motif (Fig. 1a). The presence of Siah-degron motifs indicate towards the likelihood of a molecule to be a Siah-substrate [23, 24]. Therefore, our observation pointed to the potential of MRCKβ being a Siah2-substrate. LR_PPI prediction [26] of Siah2-MRCKβ interaction also suggested of high probability (0.9943) of their interaction (Additional file 1: Fig. S1).

In order to assess Siah2 phosphorylation after *H. pylori* infection, MKN45 cells were challenged with 200 MOI of *H. pylori* 26695 for various durations. From the western blot results, enhanced Siah2 level as well as increase in P-Ser-Siah2 and P-Thr-Siah2 were noticed in *H. pylori*-infected cells (Fig. 1b), reaching optimal levels at 12 h post infection. Siah2 phosphorylation was optimal at 12 h of *H. pylori* infection at 200 MOI (Fig. 1c). To assess *cag* PAI-dependence on Siah2 Ser/Thr phosphorylation, we infected MKN45 cells with MOI 200 of the *cag* PAI + *H. pylori* strain 26695 or the *cag* PAI - *H. pylori* strain 8–1 for 12 h. Siah2 phosphorylation was induced by both the strains as detected by western blot (Fig. 1d).

### MRCKβ protein is downregulated in *H. pylori*-infected GECs

**Fig. 2:**
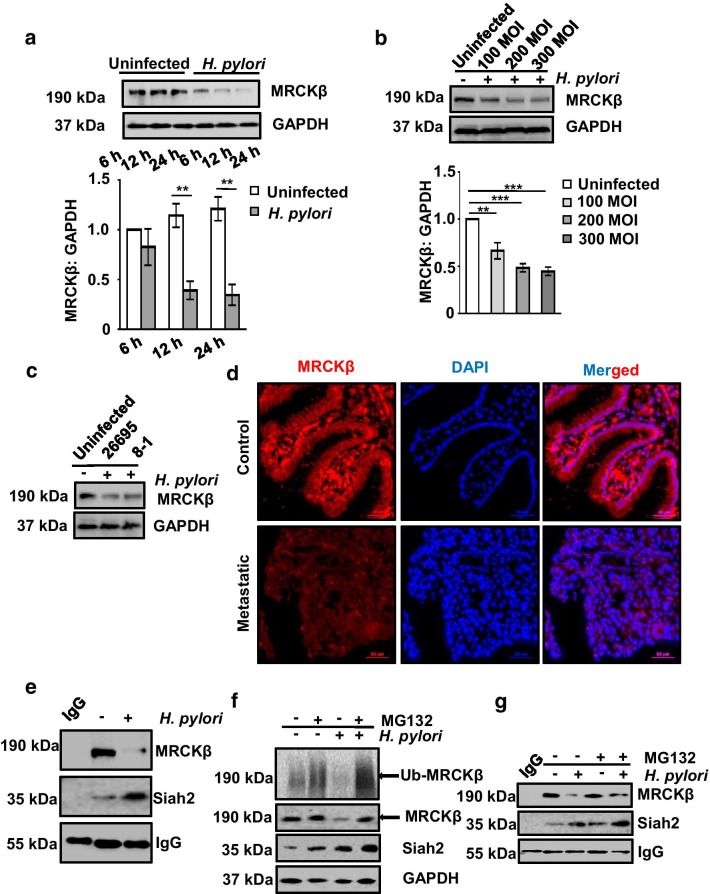
MRCKβ is decreased in *H. pylori-*mediated GC and interacts with Siah2. **a** Western blot of whole cell extracts from uninfected and *H. pylori*-infected MKN45 cells (at 6, 12, and 24 h of infection with 200 MOI of *H. pylori* 26695) showing protein level of MRCKβ. GAPDH is a loading control. Bar graph clearly indicates a time-dependent decrease of MRCKβ after *H. pylori* infection. Two-way ANOVA followed by Tukey’s post hoc analysis is used to determine statistical significance. **b** Western blotting of total cell lysate from uninfected and *H. pylori-*infected (at MOI 100, 200 and 300) MKN45 cells showing MRCKβ protein. GAPDH is used as a loading control. Graph represents decrease of MRCKβ protein as compared to uninfected control. One-way ANOVA is used to determine statistical significance. **c** A representative western blot of infected (200 MOI for 12 h of *H. pylori* 26695 or 8–1 strain) or uninfected MKN45 cells showing MRCKβ protein level (n = 3). GAPDH is used as a loading control. **d** A representative (n = 9) immunofluorescence micrograph of human metastatic GC biopsy tissue samples showing the status of MRCKβ. Tissues are sectioned at 5 μm thickness. Images are captured using 40X objective and scale bars = 50 μm. **e** Western blot of whole cell lysate from uninfected and infected (*H. pylori* at 200 MOI for 12 h) MKN45 cells subjected to co-immunoprecipitation using Siah2 antibody reveals that Siah2 interacts with MRCKβ. IgG band is used to indicate equal loading. **f** A representative western analysis indicating ubiquitination status of MRCKβ from uninfected or infected (*H. pylori* at 200 MOI for 12 h) and 50 µM MG132-treated MKN45 cells. Reprobed bands of MRCKβ, Siah2 and GAPDH. **g** Western blotting of total cell lysates from uninfected or infected (*H. pylori* at 200 MOI for 12 h) and 50 µM MG132-treated MKN45 cells subjected to co-immunoprecipitation using Siah2 antibody showing MRCKβ and Siah2 protein status. IgG band is used as the indicator of equal loading. All data are mean ± sem (n = 3). **P < 0.01, ***P < 0.001

Several kinases interacting with Siah2 get degraded by phosphorylation-mediated augmented Siah2 activity [11, 13, 14]. Necessitated by the presence of Siah-degron motifs in the MRCKβ protein sequence, examining *H. pylori-*driven regulation of the latter was imperative. The GEC MKN45 was therefore, challenged with *H. pylori* (MOI 200) for various time periods. Western blot results revealed that MRCKβ was significantly low in the infected cells at 12 h post infection as compared to the uninfected lanes (Fig. 2a). We also compared 100, 200 and 300 MOIs to find the optimal infection ratio to downregulate MRCKβ. Interestingly, at 200 MOI and 12 h of infection, MRCKβ protein level was optimally decreased in MKN45 cells as compared to the uninfected cells (Fig. 2b). In order to identify the role of *cag* PAI on MRCKβ decrease, MKN45 cells were infected with 200 MOI of *cag* PAI + *H. pylori* strain 26695 and *cag* PAI - *H. pylori* strain 8-1 at 200 MOI. Analysis of western blot showed that MRCKβ downregulation was *cag* PAI-independent (Fig. 2c). To study the MRCKβ status in human metastatic GC, antral biopsy tissues were obtained from consenting individuals followed by immuno-staining with MRCKβ antibody. A profound decrease in MRCKβ protein was observed in metastatic samples as compared to their paired-controls (Fig. 2d).

MRCKβ interaction with Siah2 was confirmed by infecting MKN45 cells with *H. pylori* for 12 h at 200 MOI followed by co-immunoprecipitation using Siah2 antibody. Western blotting of the immunocomplexes confirmed of MRCKβ-Siah2 interaction and indeed, decreased MRCKβ interaction was observed in *H. pylori*-infected cells (Fig. 2e). Siah2 ubiquitinates most of its interacting partners and marks them for proteasome-mediated degradation [27, 28]. To assess the ubiquitination status of MRCKβ in infected cells, MKN45 cells were treated with 50 µM MG132, a proteasomal inhibitor, along with *H. pylori* infection for 12 h at 200 MOI. Western blotted membranes were probed with ubiquitin antibody to detect formed ubiquitin aggregates. MG132 treatment rescued ubiquitinated proteins in the infected cells (Fig. 2f). Reprobing of the band for MRCKβ confirmed about the ubiquitination of the protein in the infected GECs. Further, we wanted to assess the interaction status of MRCKβ-Siah2 after MG132 treatment. For this, MKN45 cells were treated with 50 µM of MG132 followed by *H. pylori* infection for 12 h or were left untreated. Immunoprecipitation of whole cell lysates were performed using Siah2 antibody and the western blotting revealed less MRCKβ decrease in MG132-treated infected cells (Fig. 2g).

### Siah2 degrades MRCKβ in *H. pylori*-infected GECs

**Fig. 3:**
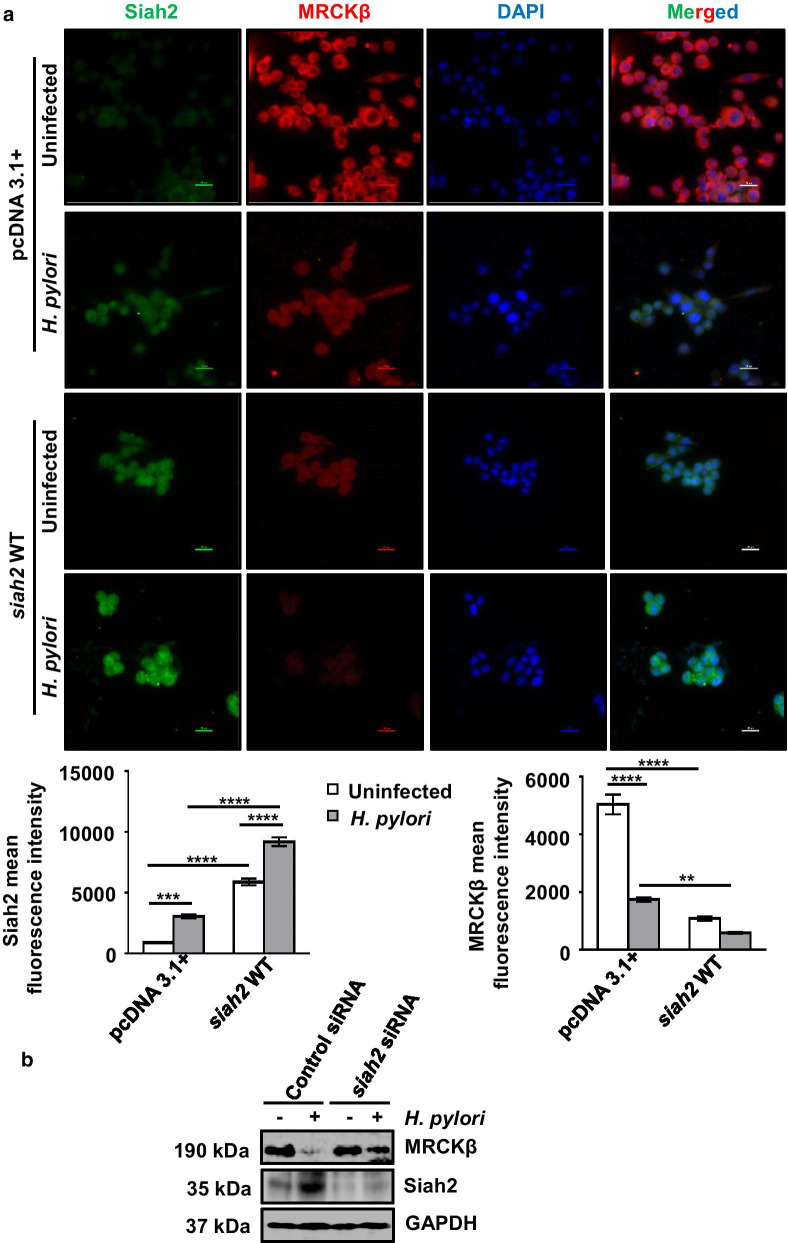
MRCKβ is degraded by Siah2. **a** Representative (n = 3) fluorescence microscopy of pcDNA3.1+ and *siah2* WT stably-expressing MKN45 cells infected with *H. pylori* (200 MOI for 12 h). Images are captured using 60X objective and scale bars represent 20 μm. Bar graphs represent mean fluorescence intensity of Siah2 and MRCKβ obtained from cells of three independent experiments. Two-way ANOVA is used to determine statistical significance. All data are mean ± sem (n = 3). **P < 0.01, ***P < 0.001, ****P < 0.0001. **b** A representative western blot of control siRNA and *siah2* siRNA-transfected MKN45 cells followed by 12 h *H. pylori* infection showing MRCKβ and Siah2 protein levels. GAPDH is used as a loading control

To investigate whether MRCKβ decrease was correlated with Siah2 increase, we infected pcDNA3.1+ and *siah2* WT MKN45 stable cells with *H. pylori*. Immunofluorescence microscopy revealed that MRCKβ was downregulated in *H. pylori*-infected cells and this was further decreased in *siah2* WT stable cells (Fig. 3a). In order to confirm Siah2-dependent MRCKβ protein decrease, we transfected MKN45 cells with siRNA of *siah2* for 36 h and infected with *H. pylori for* 12 h. Western blotting revealed that MRCKβ band was rescued after suppression of *siah2,* thus confirming about the role of Siah2 in the downregulation of MRCKβ (Fig. 3b).

### Siah2 is phosphorylated and stabilized by MRCKβ

**Fig. 4:**
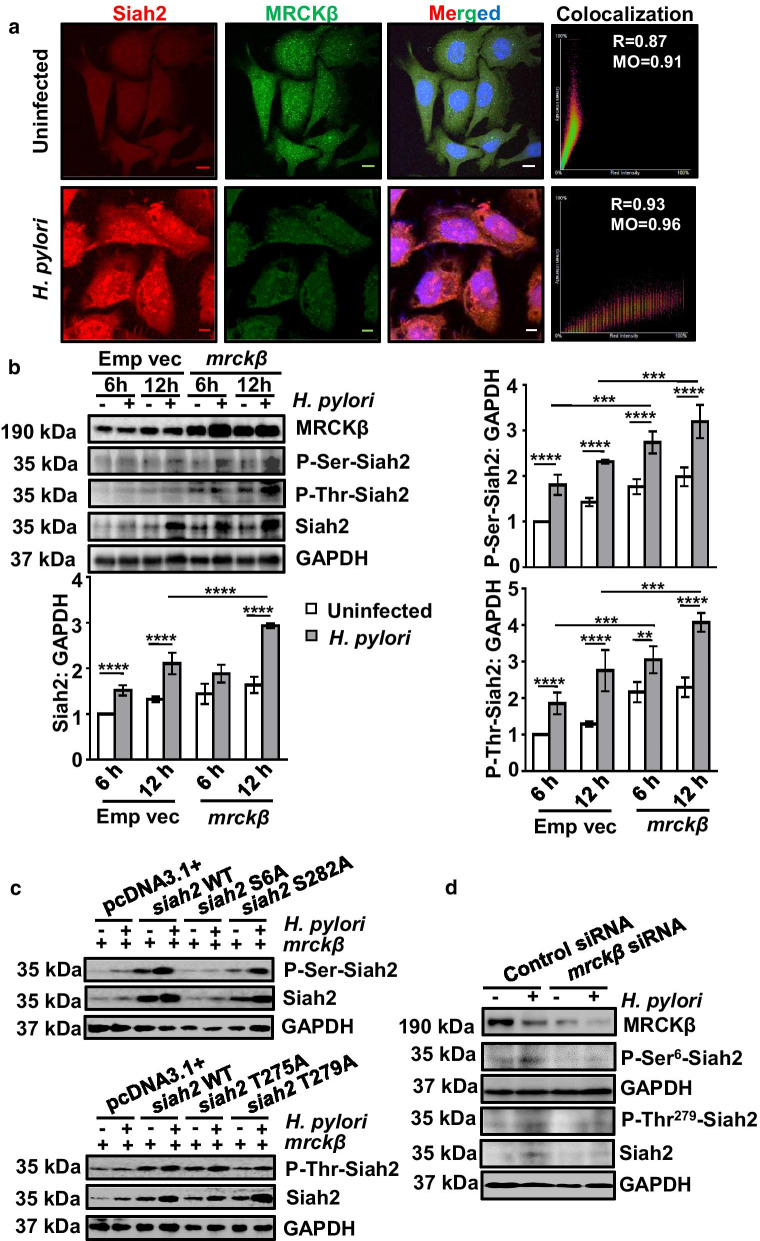
MRCKβ potentiates Siah2 phosphorylation in GECs after *H. pylori* infection. **a** Confocal images (n = 3) of uninfected and *H. pylori*-challenged (200 MOI for 12 h) AGS cells representing subcellular localization of Siah2 and MRCKβ proteins. Individual DAPI-stained panels showing nuclear staining are not shown due to constraint of space but the merged panels include DAPI. Scale bar represents 5 μm. Scatter plots generated by using NIS AR software represent co-localization of MRCKβ and Siah2 along with their Pearson's correlation (R) and Mander’s overlap (MO) values. **b** A representative (n = 3) western blot of whole cell lysates of MKN45 cells transfected with pcDNA3.1+ and *mrckβ* and infected with *H. pylori* showing MRCKβ, P-Ser-Siah2, P-Thr-Siah2 and Siah2 protein status. GAPDH = loading control. Bar graphs indicate increase of P-Ser-Siah2, P-Thr-Siah2 and Siah2 after *mrckβ* overexpression followed by 12 h of *H. pylori* infection. Two-way ANOVA followed by Tukey’s post hoc analysis are performed to evaluate statistical significance. Data are mean ± sem (n = 3). **P < 0.01, ***P < 0.001, ****P < 0.0001. **c** Representative western blot (n = 3) of pcDNA3.1+, *siah2* WT and Siah2 phospho-null mutant-expressing MKN45 stable cells transfected with *mrckβ* followed by *H. pylori* infection showing P-Ser-Siah2 and Siah2 (above) and P-Thr-Siah2 and Siah2 (below). GAPDH is the loading control. **d** Representative western blot (n = 3) from whole cell lysate of MKN45 cells transfected with either control or *mrckβ* siRNA followed by *H. pylori* infection indicating MRCKβ, P-Ser^6^-Siah2, P-Thr^279^-Siah2 and Siah2 proteins. GAPDH is kept as a loading control

To study the colocalization of MRCKβ with that of Siah2, we infected AGS cells with *H. pylori* and cells were processed for immunostaining of Siah2 and MRCKβ. Confocal microscopy showed that Siah2 level was increased with a concomitant decrease of MRCKβ. Analysis of the merged image indicated the degree of colocalization between Siah2 and MRCKβ (Fig. 4a).

To identify the role of MRCKβ in Siah2 phosphorylation in MKN45 cells, cells were transfected with either the empty vector (pEGFP-N1) or WT *mrckβ* for 36 h followed by *H. pylori* infection for 6 h or 12 h. Western blotting and graphical presentation with statistical analysis confirmed that P-Ser-Siah2, P-Thr-Siah2 increased significantly along with Siah2 after *mrckβ* overexpression and 12 h *H. pylori*-infection (Fig. 4b), but not at 6 h, when Siah2 did not yet reach its optimal level. In order to identify the probable Ser/Thr phosphorylation residues of Siah2, NetPhos3.0 software [29] was used. Among the predicted Ser/Thr residues of Siah2 with high probability of phosphorylation are shown in Additional file 1: Fig. S2A. Out of the probable phosphorylation residues, phospho-Ser^6^-Siah2 was detected in HeLa cell phosphoproteome [30] but its significance has never been studied. Similarly, the effect of Siah2 phosphorylation at Thr^275^, Thr^279^ and Ser^282^ are also not known. The schematic depiction of the domain arrangement of Siah2 indicates the position of these residues (Additional file 1: Fig. S2B).

In order to study the impact of phosphorylation at these residues, we generated phosphorylation-null mutants of Siah2 (S6A, T275A, T279A and S282A) by site-directed mutagenesis. To consistently study phosphorylation events, we generated pcDNA3.1+, WT Siah2 and Siah2 S6A, T275A, T279A and S282A phospho-null mutant stably-expressing MKN45 cells. In order to identify the phosphorylated residues of Siah2, MKN45 pcDNA3.1+, *siah2* WT, Siah2 phospho-null mutant-expressing stable cells were transfected with *mrckβ* and were infected with *H. pylori* or were left uninfected*.* Siah2 phosphorylation was increased in all *H. pylori*-infected cells except for S6A as detected by western blotting. In the case of phospho-null mutant T279A-expressing cells, the increase in phosphorylation post-infection was lesser than the WT and T275A-infected cells indicating that Thr^279^ was the prime site for Siah2 phosphorylation in infected GECs (Fig. 4c). The representative data (n = 3) also suggested that Siah2 phosphorylation at Ser^6^ and Thr^279^ has role in Siah2 stability. To understand the role of *mrckβ* suppression on Siah2 stability and phosphorylation, *mrckβ* siRNA or control siRNA were transfected in MKN45 cells for 36 h and were challenged with *H. pylori* for 12 h. Western blot analysis using custom-prepared phospho-specific Siah2 antibodies (P-Ser^6^-Siah2 and P-Thr^279^-Siah2) showed abrogation of Siah2 phosphorylation as well as Siah2 downregulation by *mrckβ* suppression (Fig. 4d). These results indicated that MRCKβ-mediated Siah2 phosphorylation at Ser^6^ and Thr^279^ contributed in Siah2 stability.

### *H. pylori* infection remarkably enhances Siah2 phosphorylation at Ser^6^ and Thr^279^ in GECs and in metastatic GC biopsies

**Fig. 5:**
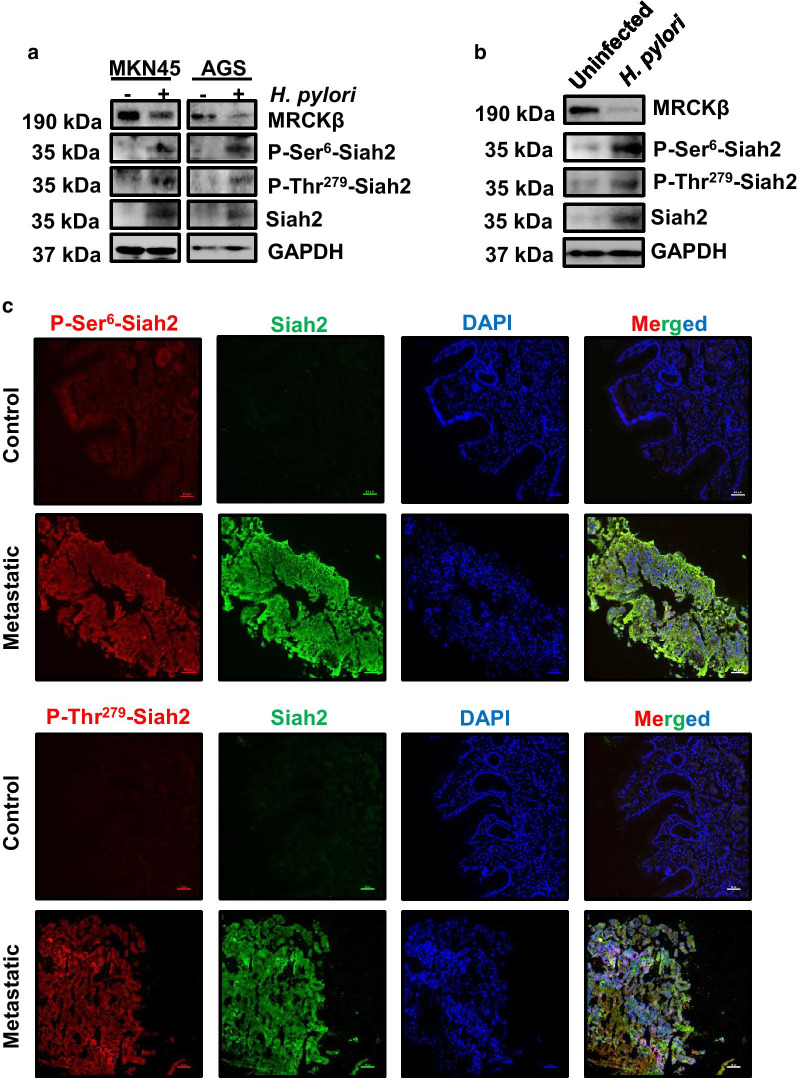
Increase in P-Ser/Thr-Siah2 in GECs and in human GC samples. **a** A representative western blot (n = 3) of uninfected and infected (200 MOI *H. pylori*, 12 h) MKN45 and AGS cells showing enhanced P-Ser^6^-Siah2, P-Thr^279^-Siah2 and Siah2 proteins. GAPDH is the loading control. **b** Representative western blot (n = 3) of uninfected and infected (200 MOI *H. pylori*, 12 h) HFE145 cells showing enhanced P-Ser^6^-Siah2, P-Thr^279^-Siah2 and Siah2 proteins. GAPDH is the loading control. **c** Immunofluorescence microscopy images of human gastric biopsy tissue (n = 9) showing the status of Siah2, P-Ser^6^-Siah2 (above) and P-Thr^279^-Siah2 (below) proteins. Tissues are sectioned at 5 μm thickness. Images are captured using 20× objective and scale bars represent 50 μm

To assess the status of Ser^6^ and Thr^279^-phosphorylated Siah2 in GECs, AGS and MKN45 cells were infected with *H. pylori* for 12 h and western blotting was performed. Results showed enhanced level of Ser^6^ and Thr^279^ phosphorylated Siah2 in infected MKN45 as well as AGS cells (Fig. 5a). Response of the immortalized but non-neoplastic GEC HFE145 to *H. pylori* infection was also investigated. For this, HFE145 cells were infected with *H. pylori* for 12 h and western blotting was performed. Enhanced level of Ser^6^ and Thr^279^-phosphorylated Siah2 protein and decreased level of MRCKβ protein were noted in *H. pylori*-infected HFE145 cells (Fig. 5b). Immunofluorescence microscopy of human metastatic GC biopsy samples showed profound increase of Siah2, P-Ser^6^-Siah2 and P-Thr^279^-Siah2 but decreased MRCKβ in metastatic GC samples as compared to their paired normal gastric tissues (Fig. 5c and Additional file 1: Fig. S3i–iii). To identify the impact of MRCKβ on Siah2 phosphorylation at Ser^6^ and Thr^279^, immunofluorescence microscopy was performed using *mrckβ*-overexpressed or empty vector-overexpressed AGS cells followed by 12 h of *H. pylori* infection. Reaffirming our previous results, representative data (n = 3) showed enhanced levels of P-Ser^6^-Siah2 and P-Thr^279^-Siah2 in *H. pylori*-infected GEC (Additional file 1: Fig. S4).

### *H. felis* infection degrades MRCKβ but enhances Siah2 and its phosphorylation in C57BL/6 mice

**Fig. 6:**
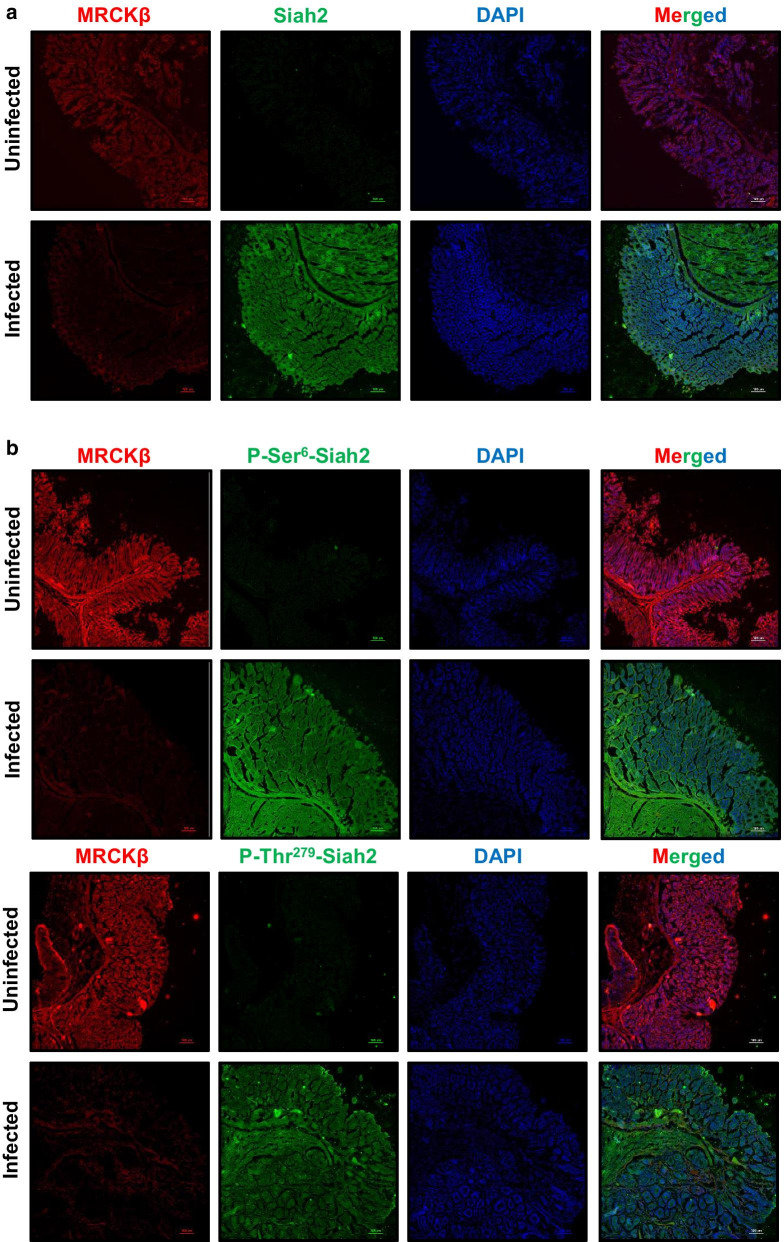
*H. felis*-infected C57BL/6 mice exhibit enhanced Siah2 phosphorylation and MRCKβ downregulation. **a** Immunofluorescence microscopy images of antral gastric tissues of uninfected and infected mice showing the status of Siah2 and MRCKβ. **b** Immunofluorescence microscopy images of antral gastric tissues of C57BL/6 mice showing the status of MRCKβ, P-Ser^6^-Siah2 and P-Thr^279^-Siah2. All tissues are  sectioned at 5 μm thickness. All images are captured using 10× objective and scale bars represent 100 μm

*H. felis*-infected C57BL/6 mice exhibit GC progression events similar to that of humans [9, 31]. Murine antral gastric samples were probed for MRCKβ and Siah2 proteins by immunofluorescence microscopy. MRCKβ was downregulated and Siah2 was upregulated in infected tissues as compared to uninfected controls (Fig. 6a). Immunofluorescence microscopy of infected murine antral gastric samples also exhibited increased P-Ser^6^–Siah2 (above) and P-Thr^279^-Siah2 (below) as compared to uninfected controls (Fig. 6b). Therefore, these in vivo findings further strengthened our in vitro results.

Taken together, data shown in Figs. 2, 3, 4, 5, 6 indicated that *Helicobacter*-mediated Siah2 phosphorylation at Ser^6^ and Thr^279^ was driven by MRCKβ but Siah2-MRCKβ interaction degraded MRCKβ.

### Phosphorylation of Siah2 promotes GC progression and invasiveness

**Fig. 7:**
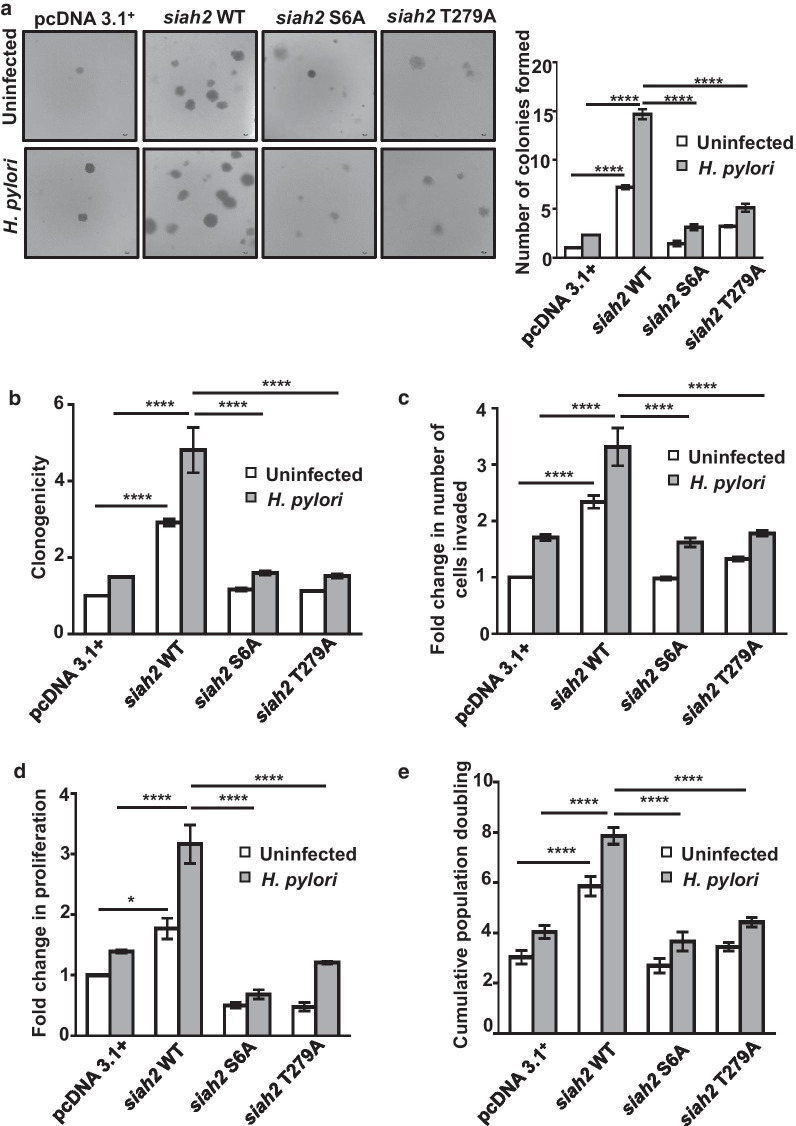
Phosphorylated Siah2 regulates GC progression. **a** Brightfield images showing colonies formed in soft agar assay (n = 3) by pcDNA3.1+, WT *siah2*, S6A and T279A *siah2* mutant-expressing MKN45 stable cells. Images are taken using 4X objective and scale bar represents 100 μm. Colonies greater than 50 μm are counted. **b** Bar graph represents change in the colony formation ability of pcDNA3.1+, WT *siah2* and Siah2 phospho-null mutant stably-expressing MKN45 cells. Results are normalized to the uninfected empty vector control. **c** Graphical representation of changes in the invasive potential of pcDNA3.1+, *siah2* WT and Siah2 phospho-null mutant-expressing AGS stable cells as assessed by the number of invaded cells. Values are normalized to uninfected empty vector control. **d** Graphical representation of change in cellular proliferation assessed by MTT assay using pcDNA3.1+, *siah2* WT and Siah2 phospho-null mutant-expressing MKN45 stable cells. **e** Graphical representation of cumulative population doubling of uninfected and *H. pylori*-infected pcDNA3.1+, *siah2* WT and Siah2 phospho-null mutant-expressing MKN45 stable cells. In all of these panels two-way ANOVA following Tukey’s post hoc analysis calculates the statistical significance. All data represent mean ± sem (n = 3), *P < 0.05, ****P < 0.0001

Siah2 and its acetylation enhances GC invasion and metastasis [7–9]. In order to investigate role of Siah2 phosphorylation on anchorage-independent growth of GEC, pcDNA3.1+, WT and phospho-null mutant *siah2*-expressing MKN45 stable cells were either left uninfected or were infected for 12 h at 200 MOI and used for soft agar assay. We found a significantly decreased number of colonies in case of Siah2 phospho-null mutants as compared to *siah2* WT, which formed maximum number of colonies after *H. pylori* infection (Fig. 7a). Anchorage-dependent growth of these stable cells were assessed. Cells were either left uninfected or were challenged with 200 MOI *H. pylori*-for 12 h. We found a significant increase in the number of colonies formed in *siah2* WT cells as compared to the empty vector and Siah2 phospho-null mutant-expressing cells (Fig. 7b). Influence of Siah2 phosphorylation on invasion was examined using pcDNA3.1+, *siah2* WT and phospho-null mutant *siah2*-expressing AGS stable cells. MKN45 cells are semi-adherent in nature and, therefore, were not used for invasion assays [8, 9]. Invasive potential of *siah2* WT stable cells was significantly higher as compared to their phospho-null counterparts (Fig. 7c). Role of Siah2 phosphorylation on cellular proliferation was assessed by MTT assay and cell population doubling assay using *H. pylori*–infected or uninfected pcDNA3.1+, *siah2* WT and Siah2 phospho-null mutant-expressed MKN45 stable cells. We found that proliferation of Siah2 phospho-null mutant cells was significantly less than *siah2* WT cells 12 h post-infection (Fig. 7d and e). Altogether, these results establish that Siah2 phosphorylation had an important role in GC progression and invasiveness.

## Discussion

Siah2 is upregulated in response to *H. pylori* infection and promotes proliferation as well as invasiveness of GECs [7, 8]. The main finding of this study is that Siah2 is phosphorylated by the Ser/Thr Kinase MRCKβ in GECs infected with *H. pylori*. Phosphorylation enhances Siah2 stability and our results reveal the importance of Siah2 phosphorylation in increasing GEC survival, carcinogenic/proliferative potential, invasiveness and anchorage-independent growth. Therefore, interference of Siah2 phosphorylation might be a useful therapeutic approach for the treatment of GC.

Dynamic phosphorylation-dephosphorylation events modulate many cellular processes. Phosphorylation of proteins often act as on–off switches for protein function. Apoptosis-regulatory proteins and proteins involved in EMT are very tightly regulated by differential phosphorylation [32–34]. Phosphorylation controls enzyme activities and phosphorylation-mediated deregulations of ubiquitinating enzymes are major threats in causing progression of cancer and degenerative diseases [35]. Phosphorylation of the E3 ubiquitin ligase Siah2 is pivotal in regulating its function [11–14]. This study has identified for the first time that Siah2 is phosphorylated at Ser^6^ and Thr^279^ residues by MRCKβ in *H. pylori*-infected GECs, human antral GC biopsy samples and *H. felis*-infected murine gastric tissues. Our results are supported by a mass spectrometry-based phosphoproteome analysis of a myelogenous leukemia cell line K562 which also identified the presence of Ser^6^-phosphorylated Siah2 in that cell but identifying the functional importance of phospho-Siah2 was not within the scope of that study [30]. Along with phosphorylation at Ser^6^ we have identified phosphorylation of Siah2 at Thr^279^ and elucidated their functional importance in GC progression. The inability to undergo phosphorylation at Ser^6^ and Thr^279^ decreases the tumorigenic functions of Siah2. Future cross-cancer studies would help us determine whether Siah2 phosphorylation is essential for carcinogenic processes.

MRCKβ belongs to a subfamily of Rho GTPases within the AGC family of kinases (i.e., protein kinases A, G and C). MRCKβ interacts with Cdc42 and regulates myosin II-regulatory light chain phosphorylation important for the regulation of actin-myosin cytoskeleton dynamics [18, 36–38]. By studies involving overexpression we show that MRCKβ is responsible for Siah2 phosphorylation. Our observation shows that although MRCKβ is a kinase phosphorylating Siah2 in *H. pylori*-challenged GECs, Siah2 is a “kiss of death” signal for MRCKβ. We find that Siah2 ubiquitinates MRCKβ. MRCKβ ubiquitination has been reported by a multiplexed mass spectrometry study in various cells and tissue samples including cancer [39]. This study is also in harmony with another observation which identified that Siah2 could regulate the cellular pool of its kinases through ubiquitination [40]. Most of the ubiquitinated proteins are targeted for proteasomal degradation [41, 42] and our results also point towards the proteasomal degradation of MRCKβ in *H. pylori*-infected GECs. Although there is no report on the status and role of MRCKβ in GC, our finding is supported by the human protein atlas information on “stomach cancer” showing that only 16.67% GC samples moderately express Cdc42BPB (MRCKβ) and the rest do not stain for the protein (v19.proteinatlas.org/ENSG00000198752-CDC42BPB/pathology) while 80% of GC tissues show expression of Siah2 protein (v19.proteinatlas.org/ENSG00000181788-SIAH2/pathology) [25]. Cdc42 is a small GTPase which is implicated in the progression of cancer via regulation of many cellular processes [31]. Cdc42 regulates tumorigenesis by modulating several kinases that are known for their tumorigenic potential [21, 41]. Since GC cell motility and polarity are highly dependent on the Rho GTPase Cdc42 [43, 44], it is imperative to study how the loss of Cdc42BPB i.e. MRCKβ is compensated in GC.

## Conclusions

In conclusion, our study shows that MRCKβ-mediated phosphorylation of Siah2 increases proliferation, survival, anchorage-independent nature and invasiveness of GECs implicating a crucial link of the mechanism with GC progression. In addition, this study establishes that phosphorylation-mediated increased stability of Siah2 and concomitant decrease in MRCKβ are important immunohistochemical characteristics of metastatic GC. As Siah2 stabilization by *H. pylori* ensures GC progression, targeting Siah2 stability looks very promising for therapeutic intervention of GC.

## Supplementary Information

**Additional file 1: Fig. S1.** Result of LR_PPI analysis showing the probability of Siah2-MRCKβ interaction. **Fig. S2.** Identification of novel phosphorylatable residues. (A) Novel Siah2 phosphorylatable residues along with their predicted scores as identified using NetPhos 3.0. (B) Sequence and multi-domain schematic depiction of human Siah2 protein. Identified highly probable phosphorylatable amino acids are underlined in the sequence and represented by vertical lines in the domain structure. **Fig. S3i.** Human gastric biopsy tissues exhibit enhanced Siah2 phosphorylation and MRCKβ downregulation. Immunofluorescence microscopy images showing the status of Siah2, P-Ser^6^-Siah2, P-Thr^279^-Siah2 and MRCKβ in Patient #1–3. Objective: 20X and scale bar represents 50 μm. **Fig. S3ii.** Human gastric biopsy tissues exhibit enhanced Siah2 phosphorylation and MRCKβ downregulation. Immunofluorescence microscopy images showing the status of Siah2, P-Ser^6^-Siah2, P-Thr^279^-Siah2 and MRCKβ in Patient #4–6. Objective: 20X and scale bar represents 50 μm. **Fig. S3iii.** Human gastric biopsy tissues exhibit enhanced Siah2 phosphorylation and MRCKβ downregulation. Immunofluorescence microscopy images showing the status of Siah2, P-Ser^6^-Siah2, P-Thr^279^-Siah2 and MRCKβ in Patient #7–9. Objective: 20X and scale bar represents 50 μm. **Fig. S4.** Phosphorylated Siah2 is enhanced upon *mrckβ* overexpression. Immunofluorescence microscopy images of AGS cells overexpressing the empty vector or *mrckβ* and infected with 200 MOI *H. pylori* for 12 h showing enhanced P-Ser^6^-Siah2 and P-Thr^279^-Siah2. Objective: 60X and scale bar represents 20 μm.

## Data Availability

The datasets used and/or analysed during the current study are available from the corresponding author on reasonable request.

## Abbreviations

*cag* PAI: Cytotoxin associated gene pathogenicity island
GC: Gastric cancer
GECs: Gastric epithelial cells
*H. pylori*: *Helicobacter pylori*
MG132: Z-Leu-Leu-Leu-al
MOI: Multiplicity of infection
MRCKβ: Myotonic dystrophy kinase-related Cdc42-binding kinase β
P-Ser^6^-Siah2: Phosphorylated Serine^6^ Siah2
P-Thr^279^-Siah2: Phosphorylated Threonine^6^ Siah2
Siah: Seven in absentia homolog
WT: Wild type
