# Supplementary material for: Helicobacter pylori-induced gastric cancer is orchestrated by MRCKβ-mediated Siah2 phosphorylation
Source: J Biomed Sci. 2021 Feb 3;28:12. doi: 10.1186/s12929-021-00710-0 (PMC7856738; doi:10.1186/s12929-021-00710-0)
Supplement: Supplementary file 1 — Additional file 1: Fig. S1. Result of LR_PPI analysis showing the probability of Siah2-MRCKβ interaction. Fig. S2. Identification of novel phosphorylatable residues. (A) Novel Siah2 phosphorylatable residues along with their predicted scores as identified using NetPhos 3.0. (B) Sequence and multi-domain schematic depiction of human Siah2 protein. Identified highly probable phosphorylatable amino acids are underlined in the sequence and represented by vertical lines in the domain structure. Fig. S3i. Human gastric biopsy tissues exhibit enhanced Siah2 phosphorylation and MRCKβ downregulation. Immunofluorescence microscopy images showing the status of Siah2, P-Ser6-Siah2, P-Thr279-Siah2 and MRCKβ in Patient #1–3. Objective: 20X and scale bar represents 50 μm. Fig. S3ii. Human gastric biopsy tissues exhibit enhanced Siah2 phosphorylation and MRCKβ downregulation. Immunofluorescence microscopy images showing the status of Siah2, P-Ser6-Siah2, P-Thr279-Siah2 and MRCKβ in Patient #4–6. Objective: 20X and scale bar represents 50 μm. Fig. S3iii. Human gastric biopsy tissues exhibit enhanced Siah2 phosphorylation and MRCKβ downregulation. Immunofluorescence microscopy images showing the status of Siah2, P-Ser6-Siah2, P-Thr279-Siah2 and MRCKβ in Patient #7–9. Objective: 20X and scale bar represents 50 μm. Fig. S4. Phosphorylated Siah2 is enhanced upon mrckβ overexpression. Immunofluorescence microscopy images of AGS cells overexpressing the empty vector or mrckβ and infected with 200 MOI H. pylori for 12 h showing enhanced P-Ser6-Siah2 and P-Thr279-Siah2. Objective: 60X and scale bar represents 20 μm. [file 12929_2021_710_MOESM1_ESM.pdf]

Supplementary Information

Helicobacter pylori-induced gastric cancer is orchestrated by MRCKβ-mediated Siah2 phosphorylation

Pragyesh Dixit[1], Shrikant B. Kokate[1,†], Indrajit Poirah[1], Debashish Chakraborty[1], Duane T. Smoot[2], Hassan Ashktorab[3], Niranjan Rout[4], Shivaram P. Singh[5], Asima Bhattacharyya[1,*]

Affiliations:[1]School of Biological Sciences, National Institute of Science Education and Research (NISER) Bhubaneswar, HBNI, P.O. Bhimpur-Padanpur, Via Jatni, Dist. Khurda 752050, Odisha, India.[2]Department of Medicine, Meharry Medical Center, Nashville, TN 37208, USA. [3]Department of Medicine, Howard University, Washington, DC 20060, USA. [4]Department of Pathology, Acharya Harihar Post Graduate Institute of Cancer, Cuttack 753007, Odisha, India. [5]Department of Gastroenterology, SCB Medical College, Cuttack 753007, Odisha, India.

Current affiliation: [†]Institute of Biotechnology, University of Helsinki, P.O. Box 56, 0014, Helsinki, Finland.

Correspondence: *School of Biological Sciences, National Institute of Science Education and Research (NISER), HBNI, P.O. Bhimpur-Padanpur, Via Jatni, Dist. Khurda 752050, Odisha, India. Tel: +91-674-2494210, e-mail: asima@niser.ac.in

>SIAH2
MSRPSSTGPSANKPCSKQPPPQPQHTPSPAAPPAAATISAAGPGSSAVPAAAAVISGPG
GGGGAGPVSPQHHELTSLFECPVCFDYVLPPILQCQAGHLVCNQCRQKLSCCPTCRGA
LTPSIRNLAMEKVASAVLFPCKYATTGCSLTLHHTEKPEHEDICEYRPYSCPCPGASCKW
QGSLEAVMSHLMHAHKSITTLQGEDIVFLATDINLPGAVDWVMMQSCFGHHFMLVLEKQ
EKYEGHQQFFAIVLLIGTRKQAENFAYRLELNGNRRRLTWEATPRSIHDGVAAAIMNSDC
LVFDTAIAHLFADNGNLGINVTISTCCP
>MRCKβ
MSAKVRLKKLEQLLLDGPWRNESALSVETLLDVLVCLYTECSHSALRRDKYVAEFLEWA
KPFTQLVKEMQLHREDFEIIKVIGRGAFGEVAVVKMKNTERIYAMKILNKWEMLKRAETA
CFREERDVLVNGDCQWITALHYAFQDENHLYLVMDYYVGGDLLTLLSKFEDKLPEDMAR
FYIGEMVLAIDSIHQLHYVHRDIKPDNVLLDVNGHIRLADFGSCLKMNDDGTVQSSVAVG
TPDYISPEILQAMEDGMGKYGPECDWWSLGVCMYEMLYGETPFYAESLVETYGKIMNH
EERFQFPSHVTDVSEEAKDLIQRLICSRERRLGQNGIEDFKKHAFFEGLNWENIRNLEAP
YIPDVSSPSDTSNFDVDDDVLRNTEILPPGSHTGFSGLHLPFIGFTFTTESCFSDRGSLK
SIMQSNTLTKDEDVQRDLEHSLQMEAYERRIRRLEQEKLELSRKLQESTQTVQSLHGSS
RALSNSNRDKEIKKLNEEIERLKNKIADSNRLERQLEDTVALRQEREDSTQRLRGLEKQH
RVVRQEKEELHKQLVEASERLKSQAKELKDAHQQRKLALQEFSELNERMAELRAQKQK
VSRQLRDKEEEMEVATQKVDAMRQEMRRAEKLRKELEAQLDDAVAEASKERKLREHSE
NFCKQMESELEALKVKQGGRGAGATLEHQQEISKIKSELEKKVLFYEEELVRREASHVL
EVKNVKKEVHDSESHQLALQKEILMLKDKLEKSKRERHNEMEEAVGTIKDKYERERAML
FDENKKLTAENEKLCSFVDKLTAQNRQLEDELQDLAAKKESVAHWEAQIAEIIQWVSDEK
DARGYLQALASKMTEELEALRSSSLGSRTLDPLWKVRRSQKLDMSARLELQSALEAEIR
AKQLVQEELRKVKDANLTLESKLKDSEAKNRELLEEMEILKKKMEEKFRADTGLKLPDFQ
DSIFEYFNTAPLAHDLTFRTSSASEQETQAPKPEASPSMSVAASEQQEDMARPPQRPSA
VPLPTTQALALAGPKPKAHQFSIKSFSSPTQCSHCTSLMVGLIRQGYACEVCSFACHVS
CKDGAPQVCPIPPEQSKRPLGVDVQRGIGTAYKGHVKVPKPTGVKKGWQRAYAVVCDC
KLFLYDLPEGKSTQPGVIASQVLDLRDDEFSVSSVLASDVIHATRRDIPCIFRVTASLLGA
PSKTSSLLILTENENEKRKWVGILEGLQSILHKNRLRNQVVHVPLEAYDSSLPLIKAILTAAI
VDADRIAVGLEEGLYVIEVTRDVIVRAADCKKVHQIELAPREKIVILLCGRNHHVHLYPWS
SLDGAEGSFDIKLPETKGCQLMATATLKRNSGTCLFVAVKRLILCYEIQRTKPFHRKFNEI
VAPGSVQCLAVLRDRLCVGYPSGFCLLSIQGDGQPLNLVNPNDPSLAFLSQQSFDALCA
VELESEEYLLCFSHMGLYVDPQGRRARAQELMWPAAPVACSCSPTHVTVYSEYGVDVF
DVRTMWVQTIGLRRIRPLNSEGTLNLLNCEPPRLIYFKSKFSGAVLNVPDTSDNSKKQML
RTRSKRRFVFKVPEEERLQQRREMLRDPELRSKMISNPTNFNHVAHMGPGDGMQVLM
DLPLSAVPPSQEERPGPAPTNLARQPPSRNKPYISWPSSGGSEPSVTPLRSMSDPDQ
DFDKEPDSDSTKHSTPSNSSNPSGPPSPNSPHRSQLPLEGLEQPACDT

-------Predicted interaction result of the 1st two proteins:-------------------
--
The probability for interacting of the 1st two proteins: 0.9943
The probability for non-interacting of the 1st two proteins: 0.0057
Decision: SIAH2 and MRCKB can interact
--

Fig. S1. Result of LR_PPI analysis showing the probability of Siah2-MRCKβ interaction.

A

Siah2 sites	Predicted score
S^{6}	0.996
T^{275}	0.986
T^{279}	0.931
S^{282}	0.992

B

1 MSRPSS^{6}TGPSANKPCSKQPPPQPQHTPSPAAPPAAATISAAGPGSSAVPA 50
51 AAAVISGPGGGGGAGPVSPQHHELTSLFECPVCFDYVLPPILQCQAGHLV 100
101 CNQCRQKLSCCPTCRGALTPSIRNLAMEKVASAVLFPCKYATTGCSLTLH 150
151 HTEKPEHEDICEYRPYSCPCPGASCKWQGSLEAVMSHLMHAHKSITTLQG 200
201 EDIVFLATDINLPGAVDWVMMQSCFGHHFMLVLEKQEKYEGHQQFFAIVL 250
251 LIGTRKQAENFAYRLELNGNRRRLT^{275}WEAT^{279}PRS^{282}IHDGVAAAIMNSDCLVFD 300
301 TAIAHLFADNGNLGINVTISTCCP 324

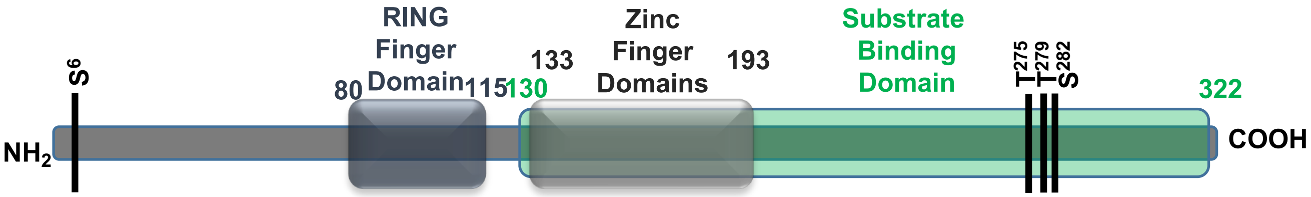

Fig. S2. Identification of novel phosphorylatable residues. (A) Novel Siah2 phosphorylatable residues along with their predicted scores as identified using NetPhos 3.0. **(B)** Sequence and multi-domain schematic depiction of human Siah2 protein. Identified highly probable phosphorylatable amino acids are underlined in the sequence and represented by vertical lines in the domain structure.

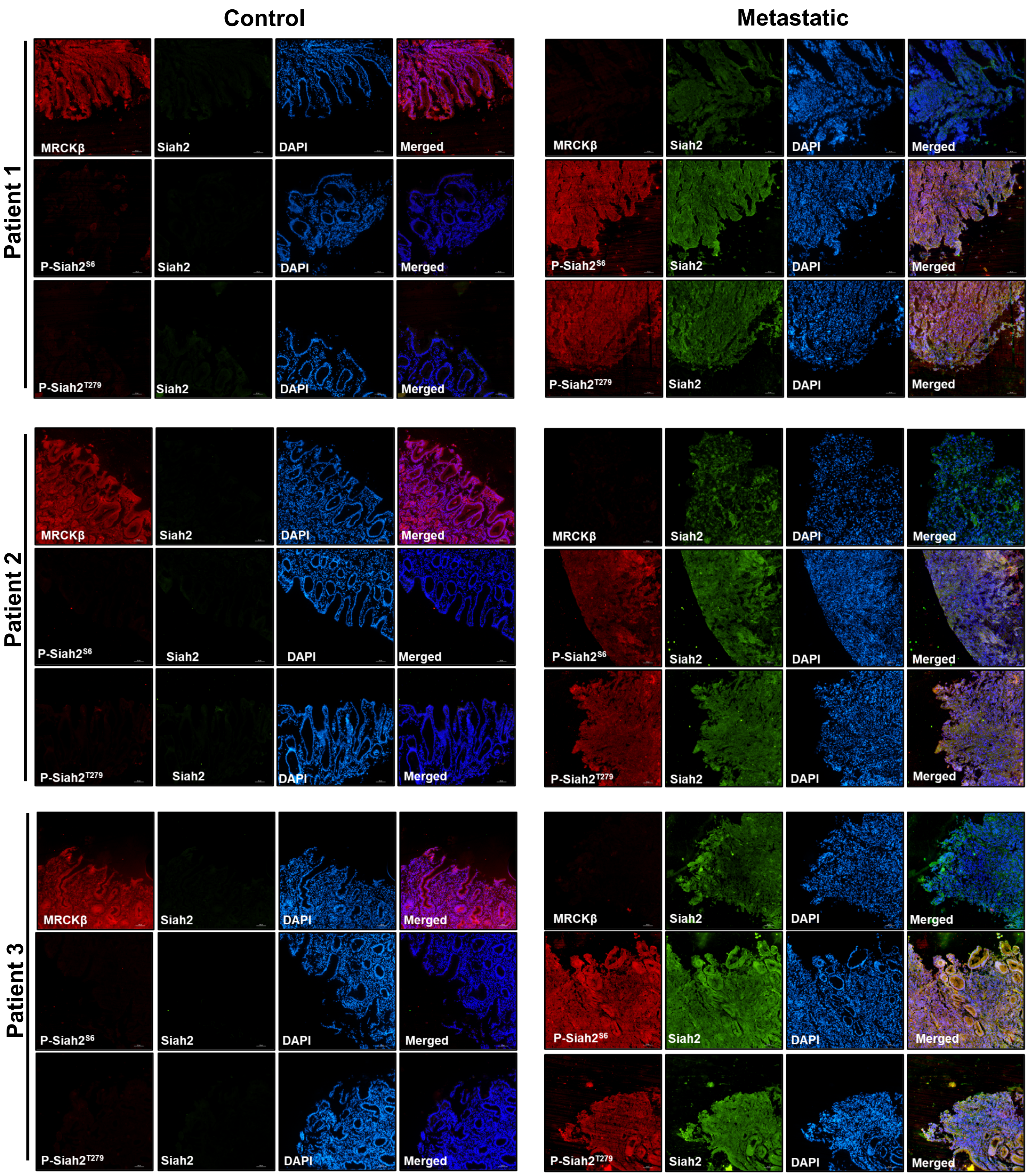

Fig. S3i. Human gastric biopsy tissues exhibit enhanced Siah2 phosphorylation and MRCKβ downregulation. Immunofluorescence microscopy images showing the status of Siah2, P-S^{6}-Siah2, P-T^{279}-Siah2 and MRCKβ in Patient #1-3. Objective: 20X and scale bar represents 50 μm.

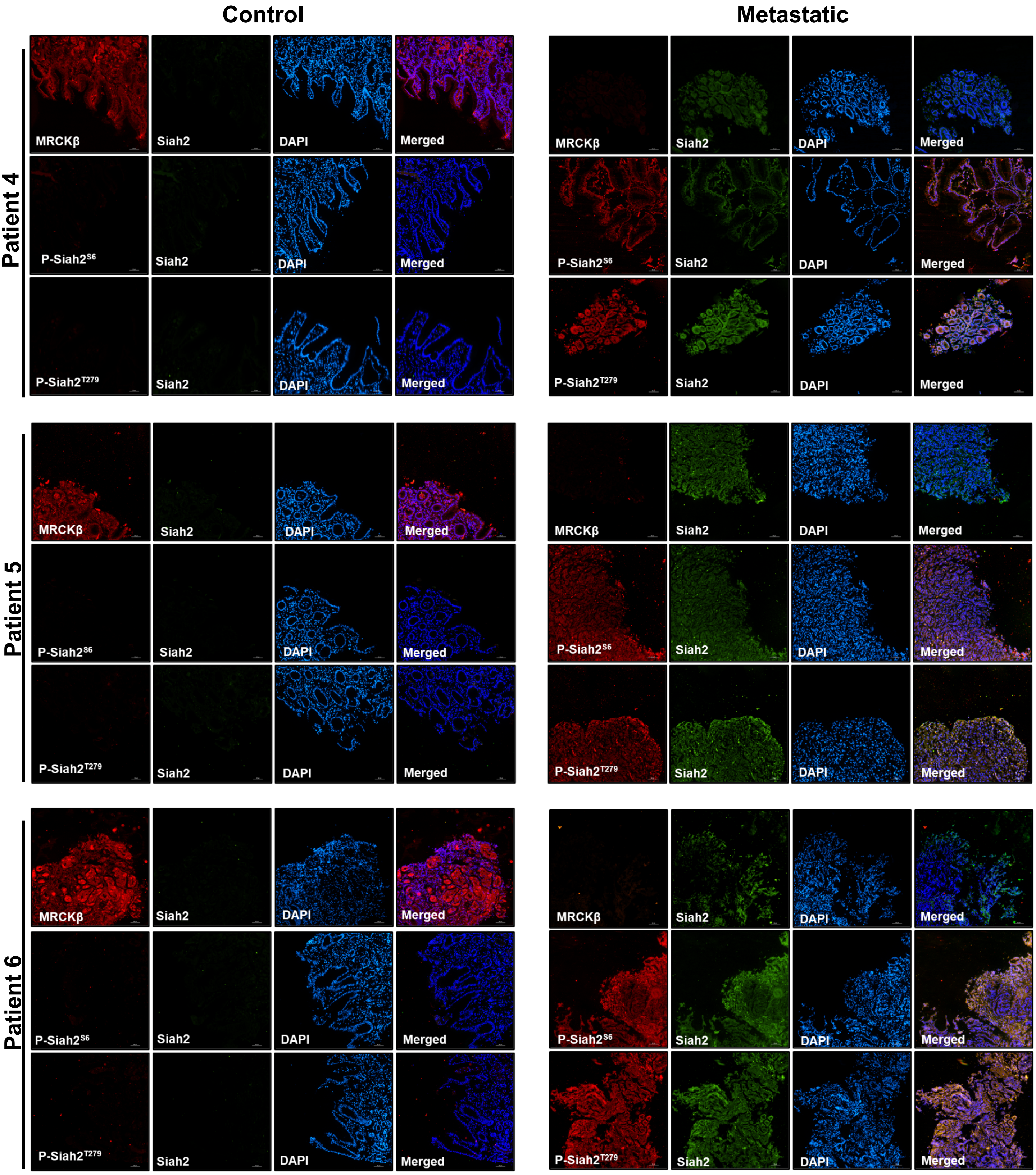

Fig. S3ii. Human gastric biopsy tissues exhibit enhanced Siah2 phosphorylation and MRCKβ downregulation. Immunofluorescence microscopy images showing the status of Siah2, P-S^{6}-Siah2, P-T^{279}-Siah2 and MRCKβ in Patient #4-6. Objective: 20X and scale bar represents 50 μm.

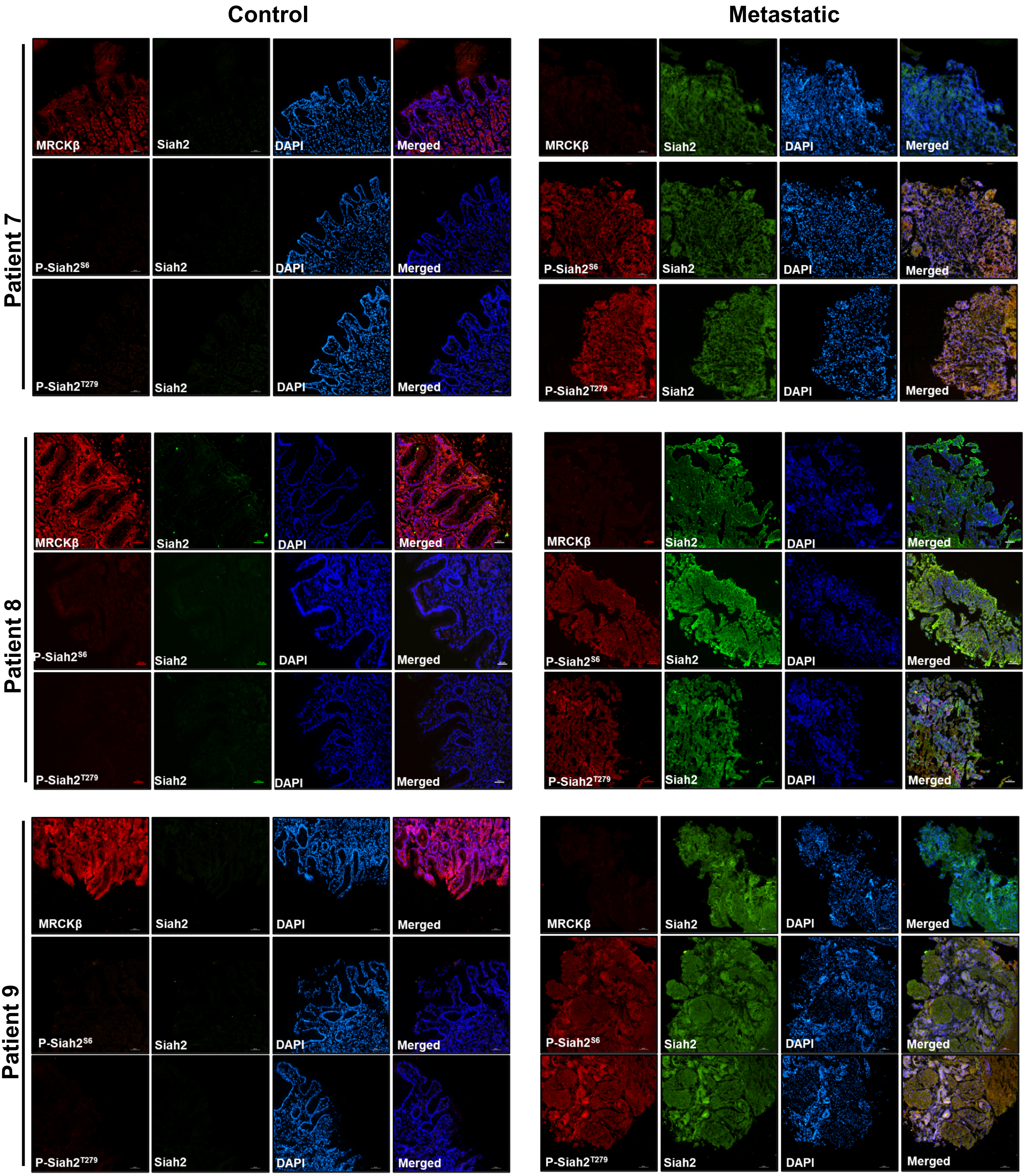

Fig. S3iii. Human gastric biopsy tissues exhibit enhanced Siah2 phosphorylation and MRCKβ downregulation. Immunofluorescence microscopy images showing the status of Siah2, P-S^{6}-Siah2, P-T^{279}-Siah2 and MRCKβ in Patient #7-9. Objective: 20X and scale bar represents 50 μm.

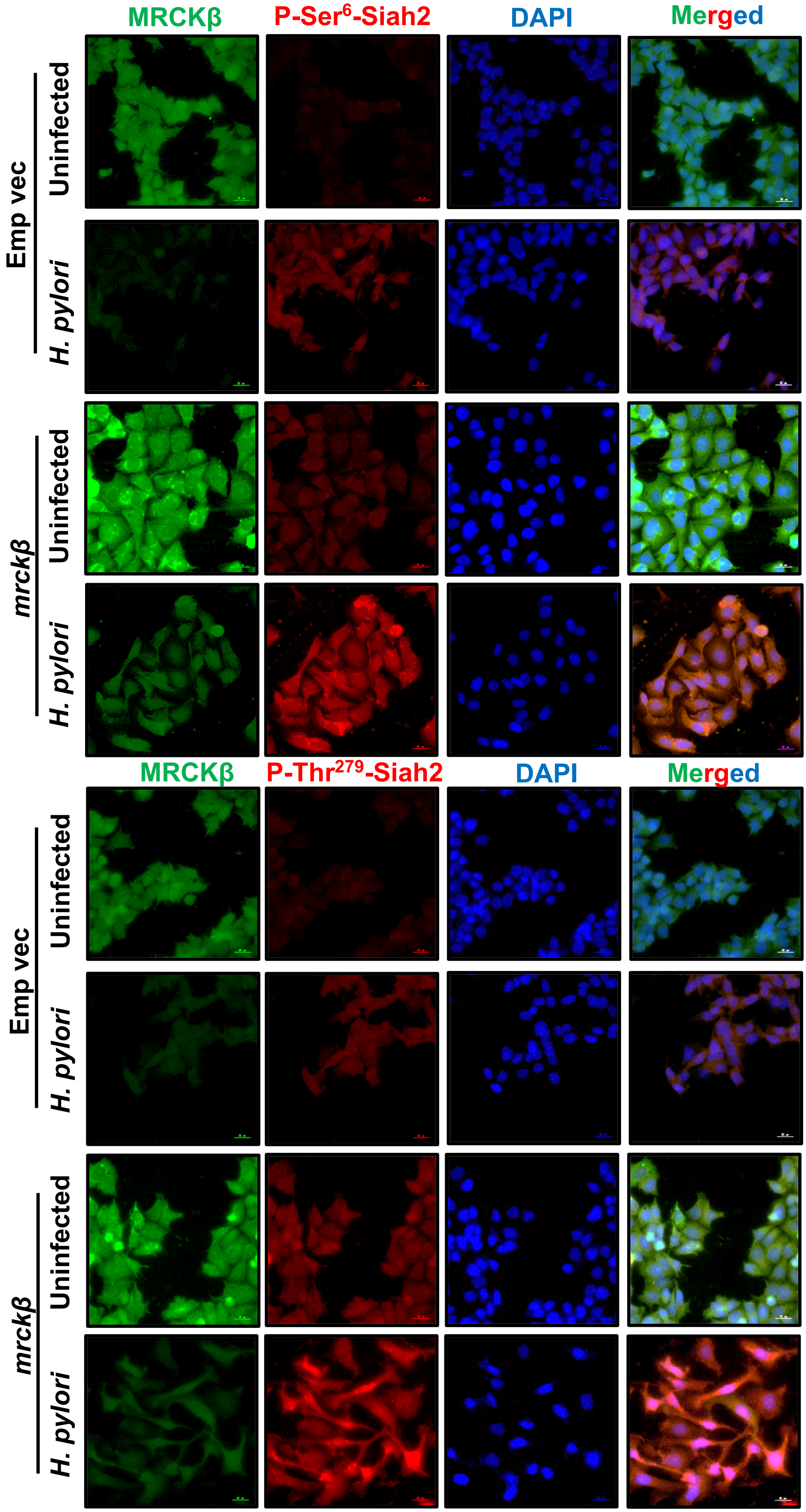

Fig. S4. Phosphorylated Siah2 is enhanced upon *mrckβ* overexpression. Immunofluorescence microscopy images of AGS cells overexpressing the empty vector or *mrckβ* and infected with 200 MOI *H. pylori* for 12 h showing enhanced P-S^{6}-Siah2 and P-T^{279}-Siah2. Objective: 60X and scale bar represents 20 μm.
